# Hand proximity differentially affects visual working memory for color and orientation in a binding task

**DOI:** 10.3389/fpsyg.2014.00318

**Published:** 2014-04-21

**Authors:** Shane P. Kelly, James R. Brockmole

**Affiliations:** Department of Psychology, University of Notre Dame, Notre Dame, INUSA

**Keywords:** visual working memory, hand posture, binding, color, orientation

## Abstract

Observers determined whether two sequentially presented arrays of six lines were the same or different. Differences, when present, involved either a swap in the color of two lines or a swap in the orientation of two lines. Thus, accurate change detection required the binding of color and orientation information for each line within visual working memory. Holding viewing distance constant, the proximity of the arrays to the hands was manipulated. Placing the hands near the to-be-remembered array decreased participants’ ability to remember color information, but increased their ability to remember orientation information. This pair of results indicates that hand proximity differentially affects the processing of various types of visual information, a conclusion broadly consistent with functional and anatomical differences in the magnocellular and parvocellular pathways. It further indicates that hand proximity affects the likelihood that various object features will be encoded into integrated object files.

## HAND PROXIMITY DIFFERENTIALLY AFFECTS WORKING MEMORY FOR COLOR AND SHAPE

The mental processing of the visual world is not independent of our physical interactions within it. A growing literature indicates that our visual experiences reflect a blending of information sensed by the eyes and motor information related to the planning and execution of various physical interactions with objects. For example, softball players who are hitting well see a bigger ball compared to those hitting poorly (Witt and Proffitt, 2005), and tennis players who return more serves successfully see the ball to be moving slower (Witt and Sugovic, 2010; see Witt, 2011 for a review). Although such group differences emerge when considering performance skill, many changes in vision can be induced within individuals by simply altering their ability to interact with objects. For example, recent studies have shown that several aspects of visual cognition are influenced by the proximity with which people hold their hands to objects being inspected (see Brockmole et al., 2013 for a review).

The links between mental processes and hand manipulations are pervasive and are observed at multiple levels of cognition. At a perceptual level, placing the hands near an object can improve visual sensitivity (Schendel and Robertson, 2004) and precision (Vishton et al., 2007). Enhancements have also been observed in one’s ability to segregate objects and backgrounds (Cosman and Vecera, 2010), to parse temporally contiguous visual events (Goodhew et al., 2013), and to recognize objects (Adam et al., 2012) near the hands. In terms of attention, objects near the hands receive attentional priority in comparison to objects located elsewhere (Reed et al., 2006, 2010; Davoli and Brockmole, 2012). Shifts of attention are also affected as attentional disengagement from items near the hands is inhibited (Abrams et al., 2008; Davoli et al., 2012a). These perceptual and attentional changes near the hands have been linked to changes in the quality of higher cognitive systems. For example, when the hands are placed near to-be-remembered objects, working memory capacity increases (Tseng and Bridgeman, 2011) and long-term memory for visual details improves (Davoli et al., 2012b). Importantly, these effects of hand proximity are not associated with possible changes in effort, comfort, response location, or hand-visibility that emerge when one places his or her hands near visual stimuli (Reed et al., 2006; Abrams et al., 2008; Davoli and Abrams, 2009; Davoli et al., 2010). Instead, we have hypothesized that these changes in memory occur because enhanced perception, focused attention, and a focus on detail allow for better processing of object properties in a region of space that has great behavioral importance (Brockmole et al., 2013).

Although the studies reviewed above catalog a variety of behavioral changes that occur near the hands, much less work has concentrated on the exact mechanisms by which they arise. One recent hypothesis links the effects of hand position to differential visual processes associated with the magnocellular and parvocellular pathways (Gozli et al., 2012; Goodhew et al., 2013, 2014). These pathways are first differentiated at the level of the retinal ganglion cells, project to different layers of the lateral geniculate nucleus, and terminate in distinct areas of visual cortex. Functionally, the parvocellular pathway processes information such as color and fine spatial details while the magnocellular pathway analyzes low-spatial frequency motion and other dynamic aspects of the world. This anatomical distinction is therefore related to a differentiation between the visual perception of form and the visual coordination of action. According to recent work, placing the hands near an object may in some sense “ready” the visual system for the processing of visually guided actions, leading to a shift, or bias, toward magnocellular processing and away from parvocellular processing. Consistent with this view, Gozli and colleagues showed that when objects appeared near the hands, detection of temporal discontinuities in object presence (a magnocellular process) improved while spatial discontinuities in object contour (a parvocellular process) diminished.

The magno/parvocellular hypothesis suggests that the effect of hand placement on object processing is nuanced, and depends on the nature of the object properties tested. The goal of the current research was to further test this account. We engaged observers in a change detection task in which they were to determine whether two sequentially presented arrays of various colored and oriented lines were the same or different. When changes were introduced, they involved either a shift in color information or a shift in orientation information. Critically, observers did not know in advance which feature might change. Hence, this task required them to memorize all aspects of the first display and to then compare the resulting visual working memory representation with a new percept. Because performance depends on the quality and contents of visual working memory (Luck and Vogel, 1997), this procedure can reveal whether one feature has representational priority over the other when hand placement is manipulated. This approach has three relative advantages over prior work. First, our method uses the same task to differentiate possible processing differences between visual features. Hence, across-task comparisons are unnecessary. Second, by randomly intermixing trial types, it becomes difficult to employ specific and different strategies on a task-by-task or even trial-by-trial basis. Third, and more importantly, our approach allows us to ask additional theoretical questions. Specifically, we can investigate whether hand posture affects one’s ability to bind visual features. Because vision acts much like a prism, splitting the processing of features such as form and color into distinct neural networks, some mechanism must reintegrate, or “bind,” these features to create a unified representation of the objects in the visual field (see Brockmole and Franconeri, 2009 for a review). If hand position differentially affects the processing of different feature categories, then it may also affect the quality of the resulting object-level representation, as some features would be better represented than others when object files are generated.

Our choice to contrast color and orientation derives from two assumptions. First, color and orientation represent distinct components of visual working memory that are each underscored by unique consolidation processes. For example, the colors of multiple objects can be consolidated in parallel (Mance et al., 2012), while the consolidation of orientation is severely limited in capacity (Woodman and Vogel, 2008; see also Stevanovski and Jolicœur, 2011; Becker et al., 2013). As such, memory for each feature may be differentially impacted by various manipulations of attentional control (in this case hand position). Second, color information is processed by the parvocellular pathway while low spatial frequency orientation information is processed by the magnocellular pathway, and, color is less relevant for potential actions (such as grasping) than is orientation. Thus, if placing the hands near an object leads to a bias in magnocellular and/or action-related processing, changes to object orientations should be best detected when objects appear near the hands while changes to object colors should be best detected when objects appear far from the hands. Alternatively, if hand proximity leads to a universal bias toward object details (cf., Davoli et al., 2012b), both color and orientation memory should be best when the stimuli appear near the hands.

## MATERIALS AND METHODS

### PARTICIPANTS

Sixty-one undergraduate students participated in exchange for course credit or monetary compensation. Three additional participants were excluded for having false alarm rates equal to or greater than hit rates. Method of remuneration did not predict performance patterns.

### STIMULI AND APPARATUS

Stimuli consisted of arrays of six lines presented on a uniform gray background. Each line measured 3 cm in length and 0.7 cm in width. The placement of each line within the array was determined by randomly selecting locations within an imaginary four by four rectangular grid. Each grid space measured 4.75 cm by 4 cm, yielding a maximum display area of 19 cm by 16 cm. The color and orientation of each line was randomly chosen. Possible colors were blue (L^*^ = 27, a^*^ = 61 b^*^ = -101), green (L^*^ = 53, a^*^ = -52 b^*^ = 49), purple (L^*^ = 34, a^*^ = 61 b^*^ = -41), and red (L^*^ = 45, a^*^ = 69 b^*^ = 60). Possible orientations were vertical, horizontal, 45° leftward slant, and 45° rightward slant. Arrays were presented on a 17 LCD monitor with a screen refresh rate of 120 Hz. Responses were made by pressing one of two response buttons. In separate conditions (described below), these buttons were affixed to either the sides of the monitor or to the table in front of the participant. Observers were seated in a stiff-backed (non-reclining) stationary chair that was placed in such as manner as to provide a constant viewing distance of approximately 40 cm with each hand position.

### DESIGN AND PROCEDURE

**FIGURE 1:**
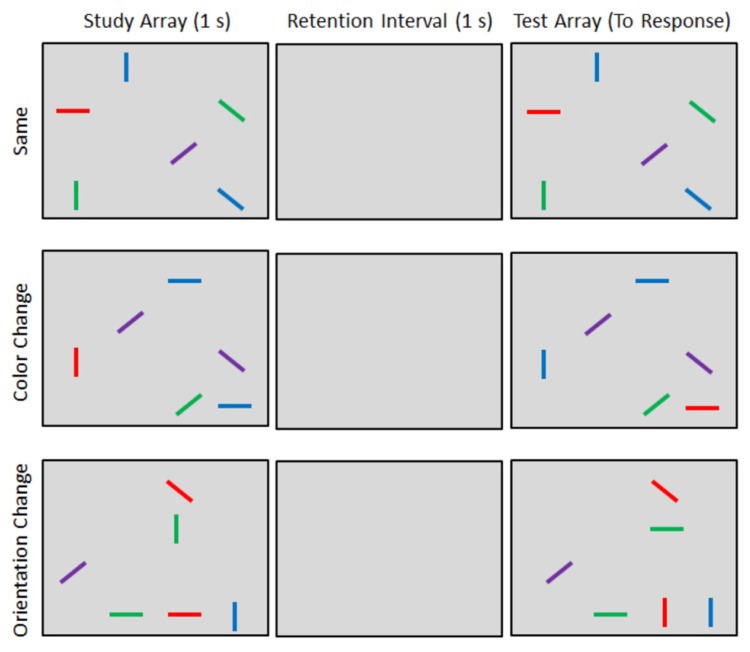
**Example stimuli and trials types. Participants indicated whether the study and test arrays were the same or different**.

On each trial, participants viewed two arrays of lines (see **Figure 1**). The study array was first presented for 1 s which was followed by a 1 s retention interval during which the screen remained blank. A second test array was then presented. The test array was either identical to the study array (**Figure 1**, top row) or incorporated changes to either the colors or orientations of the lines. On color-change trials, the colors of two randomly selected lines were swapped. For example, a study array containing a red vertical line and a blue horizontal line (**Figure 1**, middle row) could be followed by a test array that contains blue vertical and red horizontal lines (the other four lines would be unchanged from study to test). On orientation change trials, the orientations of two randomly selected lines were swapped. For example, a study array containing a green vertical line and a red horizontal line (**Figure 1**, bottom row) might be changed to include a green horizontal line and a red vertical line at test. Inducing changes to the display in this manner required observers to remember specific combinations of color, orientation, and location, rather than independent features (Wheeler and Treisman, 2002). Articulatory suppression was used throughout each trial to prevent the participants from encoding or rehearsing the stimuli verbally. At the start of each trial, two randomly selected digits were presented that the participant repeated out loud until he or she registered the change detection response.

Participants completed two blocks of 160 trials each. Within each block, 80 same trials, 40 color-change trials, and 40 orientation change trials were randomly intermixed. Across blocks, hand position was manipulated. In the hands-far block, participants placed their hands on the table top in front of them. In the hands-near block, participants’ hands were placed on the side of the monitor with their elbows resting comfortably on a cushion. In both cases, the hands remained in a stationary position (i.e., observers could not use their fingers as a mnemonic cue to store trial-by-trial information about line orientations). The order of these blocks was counterbalanced across participants. Hence, trial type (same, color-change, and orientation change) and hand position (near and far) constituted within-subjects factors.

## RESULTS

### RESPONSE TIME

Although the primary analyses of interest concerned accuracy, we submitted response times to a 2 (hand position: near or far) × 3 (trial type: same, color change, or orientation change) repeated measures analysis of variance. Neither the main effect of hand position [*F*(2,60) < 1] nor change type [*F*(2,60) = 1.38, *p* = 0.26] were reliable. Furthermore, these factors did not interact [*F*(2,60) = 1.78, *p* = 0.17]. This ensures that any observed effects in memory accuracy are not due to speed-accuracy trade-offs. Average response time was 1240 ms.

### ACCURACY

**FIGURE 2:**
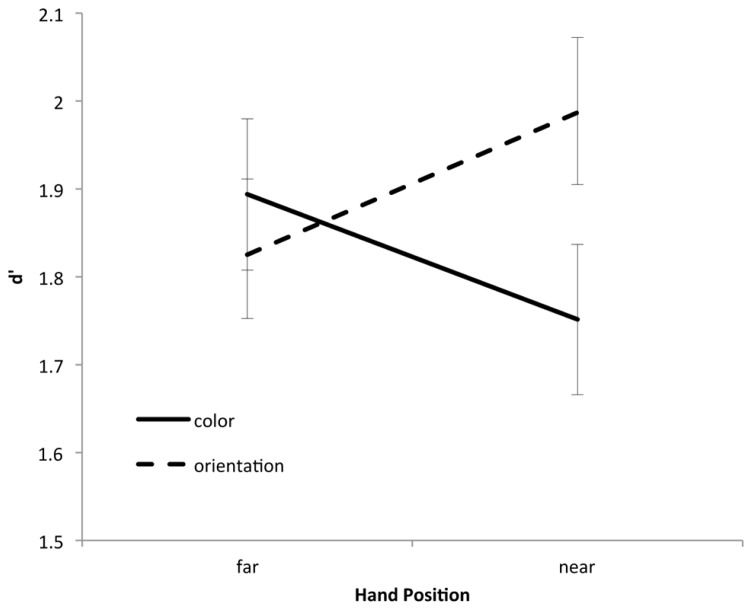
**Change detection performance as a function of hand position and change type**.

Responses were classified as hits (correct detection of a change) and false alarms. Hit rates and false alarm rates were then contrasted used a signal detection approach. Hence, d’ served as the principle measure of memory performance. Performance was analyzed in terms of a 2 (hand position: near or far) × 2 (change type: color or orientation) repeated measures analysis of variance (see **Figure 2**).

The main effects of hand position [*F*(1,60) = 1.75, *p* = 0.19] and change type [*F*(1,60) < 1] were not reliable. Importantly, however, these factors interacted [*F*(1,60) = 16.5, *p* < 0.001]. Planned comparisons showed that when the hands were far from the monitor, equal levels of performance were observed for color (*M* = 1.91) and orientation (*M* = 1.86) changes [*t*(60) < 1]. When the hands were near the monitor, however, orientation changes (*M* = 1.96) were detected better than color changes [*M* = 1.73; *t*(60) = 3.04, *p* < 0.01]. Moreover, color changes were best detected in the hands-far condition [*t*(60) = 2.05, *p* < 0.05] while orientation changes were best detected in the hands-near condition [*t*(60) = 2.18, *p* < 0.05]. Hence, placing the hands near a set of to-be-remembered objects decreases one’s ability to remember color information, but increases his or her ability to remember orientation information.

## DISCUSSION

This research considered how working memory for visual objects, defined as coherent sets of visual features (i.e., color and orientation), are maintained as a function of those objects’ proximity to the hands during viewing. Our results indicate that color memory is better when the hands are far from to-be-remembered objects while orientation memory is better when the hands are placed near those objects. This result has several implications for conceptions of the interactions between hand placement and vision as well as the structure of visual working memory itself.

Several aspects of visual perception, attention, and memory are influenced by the proximity with which people hold their hands to the objects being inspected (see Brockmole et al., 2013). Previously, we argued that these changes in cognition are adaptive as enhancements in perception, attention, and detail-oriented processing would allow for better object processing in a critical region of space near the body. Recent work, however, has suggested that these benefits may not extend to all object properties, and that the influence of hand position depends upon an anatomical distinction between the magnocellular and parvocellular visual pathways (Gozli et al., 2012; Goodhew et al., 2013, 2014). Our results support this hypothesis. If hand proximity universally affects the processing of all object features, both color and orientation memory should have been best in the near-hand condition. Instead, as the hands approached the objects, orientation processing (a magnocellular process) improved while color processing (a parvocellular process) declined. Thus, our work compliments and extends prior demonstrations supporting a magnocellular-parvocellular distinction in this arena. Our work also does so using a method in which stimulus structure and experimental task remain constant, providing important verification that prior results are unlikely to have arisen from the application of different strategies or experimenter-induced demands within different experimental tasks. So why then do these changes take place as they do? Like others, we suggest that the changes remain adaptive, but are perhaps targeted at visual information/processes most likely to be involved in the planning and execution of action.

Despite the consistency of our results with prior work noted above, our findings do provide a stark departure from some other work. Specifically, some researchers have found that placing the hands near objects increases working memory capacity – and they did so in a task where color served as the to-be-remembered feature (Tseng and Bridgeman, 2011). This is the opposite pattern from what we observed in our study. The reason for this discrepancy is not entirely clear, but differences in methodology might be important. Where improvements in color memory were observed, color was the only feature that needed to be encoded and remembered (all stimuli were colored squares). In our study, however, two features needed to be encoded and remembered (stimuli were colored lines of differing orientation). These two features may compete for processing resources [e.g., the consolidation of orientation is more severely limited in terms of capacity than is color (Becker et al., 2013), leading to a potential processing bottleneck when both features are task-relevant] and hand proximity may play a larger role in object representation under such circumstances. More work will need to be done to explore this possibility, but at a minimum the distinction suggests that a pure dissociation between magnocellular and parvocellular processing is unlikely to be an adequate explanation for hand proximity effects, at least as currently conceived.

Finally, our results shed new light on visual working memory representations when multiple features need to be bound. In order to remember the color and orientation of each line, these visual features needed to be individually processed and then bound together in memory. Our results indicate that object representations arising from these processes are sensitive to extra-perceptual factors such as hand position. When the hands were held far away from the stimuli, color, and orientation information were remembered equally well. However, when the hands were placed near the objects, color memory declined while orientation memory improved. Because both color and orientation needed to be retained on each trial, this suggests that binding in the hands-near condition was disrupted: Observers were no longer able to remember both color and shape, but were instead much more likely to retain orientation information in memory. Whether this disruption occurs early in visual processing (e.g., during stimulus encoding), represents a disturbance in reentrant processing from higher attentional processing areas (cf. Braet and Humphreys, 2009), or occurs during memory consolidation, retention, and/or retrieval remains an important question for future research.

In conclusion, we have shown that hand proximity differentially affects one’s ability to remember color and orientation information in a binding task. This result is broadly consistent with the hypothesis that magnocellular and parvocellular processes are differentially affected by hand placement, although other mechanisms likely contribute to the observed effects. The manner in which the representation of objects and their features in visual working memory is affected by extra-visual factors such as hand placement warrants further examination.

## Conflict of Interest Statement

The authors declare that the research was conducted in the absence of any commercial or financial relationships that could be construed as a potential conflict of interest.
